# Exogenous Rosmarinic Acid Application Enhances Thermotolerance in Tomatoes

**DOI:** 10.3390/plants11091172

**Published:** 2022-04-26

**Authors:** Zhiwen Zhou, Jiajia Li, Changan Zhu, Beiyu Jing, Kai Shi, Jingquan Yu, Zhangjian Hu

**Affiliations:** 1 Department of Horticulture, Zhejiang University, Hangzhou 310058, China; 21916058@zju.edu.cn (Z.Z.); 22016200@zju.edu.cn (J.L.); 12016055@zju.edu.cn (C.Z.); 12116052@zju.edu.cn (B.J.); kaishi@zju.edu.cn (K.S.); jqyu@zju.edu.cn (J.Y.); 2 Shandong (Linyi) Institute of Modern Agriculture, Zhejiang University, Linyi 276000, China; 3 Key Laboratory of Horticultural Plants Growth and Development, Ministry of Agriculture and Rural Affairs of P. R. China, Hangzhou 310058, China

**Keywords:** tomato, rosmarinic acid, thermotolerance, oxidative stress, antioxidant system, heat shock proteins, transcription regulation

## Abstract

Due to global warming, high-temperature stress has become a major threat to plant growth and development, which causes a severe challenge to food security worldwide. Therefore, it is necessary to explore the plant bioactive molecules, which could be a promising approach to strengthening plant thermotolerance. Rosmarinic acid (RA) serves as a plant-derived phenolic compound and has beneficial and health-promoting effects for human beings. However, the involvement of RA in plant stress response and the underlying molecular mechanism was largely unknown. In this study, we found that exogenous RA application conferred improved thermotolerance in tomatoes. The transcript abundance and the enzyme activity of enzymatic antioxidants, such as ascorbate peroxidase (APX), catalase (CAT), glutathione reductase (GR), and dehydroascorbate reductase (DHAR), were further promoted by RA treatment in tomato plants subjected to high-temperature stress. Moreover, RA activated the antioxidant system and modulated the cellular redox homeostasis also associated with the redox status of nonenzymatic glutathione and ascorbic acid. The results of RNA-seq data showed that transcriptional regulation was involved in RA-mediated thermotolerance. Consistently, the gene expression of several high temperature-responsive transcription factors like *HsfA2*, and WRKY family genes were substantially induced by RA treatment, which potentially contributed to the induction of heat shock proteins (HSPs). Overall, these findings not only gave a direct link between RA and plant thermotolerance but also provided an attractive approach to protecting crop plants from high-temperature damage in a global warming future.

## 1. Introduction

As sessile organisms, plants are severely threatened by a variety of abiotic stresses, such as unsuitable temperature, drought, and salinity, which cause extensive losses in agricultural production worldwide [1]. Due to increasing anthropogenic emissions, global warming dramatically affects crop growth and yield [2]. The Intergovernmental Panel on Climate Change (IPCC) reported that the average global temperature rise was expected to reach or even exceed 4.0 °C at the end of this century [3].

Tomato (*Solanum lycopersicum* L.) is the highest value and economically important vegetable crop species worldwide, and it provides a substantial source of micronutrients to humans [4]. Typically, the optimum temperature for tomato plant growth is approximately 25 °C. However, consistently high temperatures, especially over 35 °C, heavily affect all stages of tomato plants from the germination to fruit setting stages and disrupt multiple physiological and biochemical processes correlated with final yield and fruit quality. It is predicted that global warming-induced high temperatures will reduce tomato yield by as much as 70% [5]. Therefore, studying the underlying mechanism by which plants respond to high temperatures and enhancing crop thermotolerance will provide a promising avenue for maximizing agricultural production and promoting food security in the future.

Plants have evolved sophisticated thermotolerance mechanisms to combat high-temperature stress. Heat shock proteins (HSPs) and antioxidants are major functional products that are induced by high-temperature stress, and the production of HSPs and antioxidant enzymes is usually orchestrated by a heat-shock factor (Hsf)-mediated transcriptional activation [6]. As molecular chaperones, HSPs can be synthesized quickly during the early stages of high-temperature stress, and they play a crucial role in controlling protein homeostasis by renaturing numerous high temperature-denatured proteins. HSPs are generally classified into HSP100, HSP90, HSP70, HSP60, and small HSPs according to the protein molecular weight. HSP70 and HSP90 are the most abundant and important HSPs involved in thermotolerance in a variety of crops, including tomato, pepper, potato, cabbage, wheat, and tea [7]. Among multiple high temperature-responsive Hsfs, HsfA2 acts as a major transactivator during prolonged high-temperature stress by driving transcriptional memory. For instance, high temperature-induced transcript levels of *HSP70* and *HSP90* were substantially decreased in an Arabidopsis *HsfA2* knockout mutant, accompanied by reduced basal thermotolerance [8].

Photosynthesis is a well-established source of reactive oxygen species (ROS) generated from the photosynthetic electron chain, but high-temperature stress disrupts plant photosynthesis, resulting in the excessive accumulation of ROS, including superoxide anion (O_2_^•−^), hydroperoxyl radical (HO_2_^•^), hydroxyl radical (^•^OH), singlet oxygen (^1^O_2_), and hydrogen peroxide (H_2_O_2_) [9]. Consequently, excessive ROS leads to DNA, protein, lipid, and carbohydrate oxidation, which eventually amalgamate to cause oxidative stress. Plants primarily deal with oxidative stress via a complicated mechanism consisting of enzymatic and nonenzymatic antioxidants. The ROS-scavenging machinery of enzymatic antioxidants contains a series of highly efficient enzymes, such as superoxide dismutase (SOD), ascorbate peroxidase (APX), catalase (CAT), glutathione reductase (GR), glutathione peroxidase (GPX), dehydroascorbate reductase (DHAR), and monodehydroascorbate reductase (MDHAR), to control the oxidation cascades [10]. ROS are also inhibited by nonenzymatic low-molecular antioxidants, including glutathione (GSH), ascorbic acid (AsA), carotenoids, α-tocopherol, flavonoids, and phenolic compounds [11]. It is well known that a wide range of exogenous and endogenous non-enzymatic antioxidants play a positive role in plant thermotolerance. Exogenous melatonin enhanced the activity of the antioxidant system to promote the germination of rice seeds under high-temperature stress and silencing the melatonin biosynthesis gene *COMT1* in tomato plants impaired cellular redox homeostasis and aggravated high temperature-induced oxidative stress [12,13]. Similarly, the exogenous application of AsA increased the endogenous AsA content, leading to increased thermotolerance in tomato plants [14]. Thus, it might be helpful to explore plant endogenous bioactive molecules and application strategies for improving plant thermotolerance.

Rosmarinic acid (RA), named after rosemary, is a natural phenolic antioxidant and an ester of caffeic acid and 3,4-dihydroxyphenyllactic acid, which are synthesized from the amino acids l-phenylalanine and l-tyrosine, respectively [15]. RA is commonly found in species of the Boraginaceae and Lamiaceae, but it is also found in other higher plant species, including tomato [16]. RA has remarkable biological functions with a variety of applications in daily life, and many products have been prepared from RA in the food preservatives, cosmetics, and pharmaceutical industries. In particular, RA provides humans with numerous health-promoting benefits, including anti-inflammatory, anticancer, antiaging, antidiabetic, antidepressant, and antiallergic effects [17]. As a powerful antioxidant compound, it implied that RA was involved in plant tolerance to multiple abiotic stresses. For example, the endogenous RA contents, together with other phenolic compounds like caffeic acid, caftaric acid, and cinnamyl malic acid, were increased in basil (*Ocimum basilicum*) plants subjected to salt stress [18]. However, the role of RA in plant tolerance and the underlying molecular mechanisms remain largely unknown. In this study, we explored the positive role of exogenous RA in promoting thermotolerance in tomato plants, which relied on HSP induction, antioxidant system activation, and high temperature-responsive transcriptional regulation.

## 2. Results

### 2.1. Exogenous RA Treatment Increases Plant Thermotolerance in Tomato

**Figure 1:**
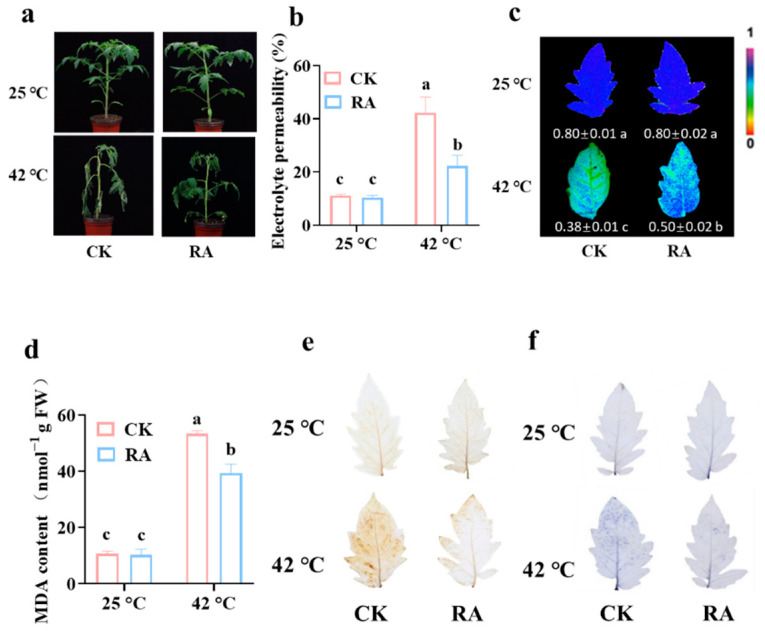
Effect of RA in tomato thermotolerance. (**a**) Representative images of tomato plants as influenced by RA and high-temperature treatment. Five-week-old tomato plants were pretreated with 2 mmol L^−1^ RA or dH_2_O control once per day for three successive days. Then, the plants were subjected to normal temperature (25 °C) or high temperature (42 °C) for 12 h. (**b**) The electrolyte leakage of tomato leaves after 12 h of different temperature treatments. (**c**) The representative leaf images show the Fv/Fm value after 12 h of different temperature treatments. The color gradient scale on the right indicates the magnitude of the fluorescence signal represented by each color. (**d**) the MDA content in tomato leaves after 12 h of different temperature treatments. (**e**) Representative images of H_2_O_2_ accumulation as determined by DAB staining. (**f**) Representative images of O_2_^•−^ accumulation as determined by NBT staining. The data presented in (**b**–**d**) are the mean values ± SD, *n* = 3. Statistically significant differences between treatments (*p* < 0.05, Tukey’s test) are shown by different letters.

To explore the role of RA in plant resistance to high temperature, tomato plants were exogenously sprayed with 2 mmol L^−1^ RA, or H_2_O as a control before different temperature treatments. After 48 h of different temperature treatments, we found that there were no differences between tomato plants pretreated with RA and H_2_O under normal temperature. However, RA effectively alleviated the leaf wilting caused by high temperatures in tomato plants (Figure 1a). Consistent with the leaf wilting phenotype, high temperature-induced electrolyte leakage could be significantly reduced by exogenous RA treatment (Figure 1b). Plant photosynthesis is quite sensitive to temperature stress. To explore whether RA played a protective effect on photosynthesis during high-temperature stress, we measured the maximum quantum efficiency of photosystem II (Fv/Fm). As shown in Figure 1c, exogenous RA treatment reduced the decline in the Fv/Fm values caused by high-temperature stress compared with dH_2_O control treatment, which implied RA effectively protected the photosynthetic performance of PSII from high temperature-induced damage.

High temperature accelerates the oxidation of cell membrane lipids, aggravating the MDA formation and ROS accumulation, which eventually causes oxidative damage and cell dysfunction [19]. To explore whether RA alleviates high temperature-induced membrane lipid peroxidation, we quantified the MDA content in tomato leaves in response to high temperature. Compared with the control pretreatment, exogenous RA application significantly reduced the MDA accumulation in tomato plants subjected to high temperatures (Figure 1d). Consistently, we evaluated ROS accumulation through DAB and NBT staining and found that RA largely inhibited high temperature-induced H_2_O_2_ and O_2_^•−^ contents, respectively (Figure 1e,f). Taken together, the above results indicated that RA effectively reduced the accumulation of harmful cellular oxidation products in tomato plants under high-temperature stress and enhanced plant thermotolerance.

### 2.2. Exogenous RA Treatment Activates Antioxidant System in Tomato Plants Subjected to High Temperature

**Figure 2:**
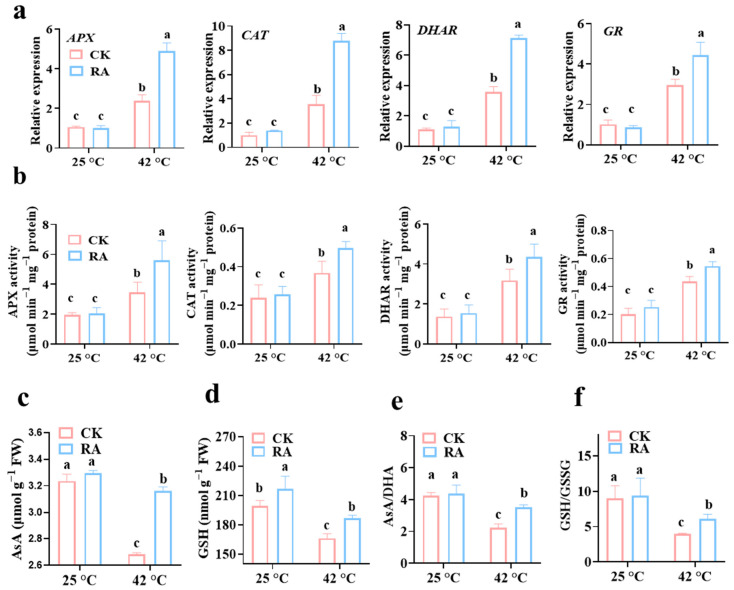
RA treatment activates the antioxidant system in tomato plants during high-temperature stress. (**a**) Effects of RA treatment on transcript abundance of antioxidant enzyme-encoding genes in tomato plants subjected to normal temperature (25 °C) or high temperature (42 °C) for 1 h. APX encodes an ascorbate peroxidase, CAT encodes a catalase, DHAR encodes a dehydroascorbate reductase, and GR encodes glutathione reductase. (**b**) Effects of RA treatment on enzyme activity of antioxidant enzymes in tomato plants subjected to different temperature conditions for 6 h. (**c**) Effects of RA treatment on AsA content in tomato plants subjected to different temperature conditions for 6 h. (**d**) Effects of RA treatment on GSH content in tomato plants subjected to different temperature conditions for 6 h. (**e**) Effects of RA treatment on AsA/DHA ratio in tomato plants subjected to different temperature conditions for 6 h. (**f**) Effects of RA treatment on GSH/GSSG ratio in tomato plants subjected to different temperature conditions for 6 h. The data presented are the mean values ± SD, *n* = 3. Statistically significant differences between treatments (*p* < 0.05, Tukey’s test) are shown by different letters.

During evolution, plants have developed a sophisticated defense system that serves to alleviate the adverse effects of temperature-induced stress. The activation of antioxidant enzymes such as ascorbate peroxidase (APX), catalase (CAT), dehydroascorbate reductase (DHAR), and glutathione reductase (GR) in plants limited or scavenged ROS accumulation, which mitigated the damages caused by temperature-induced oxidative stress [20]. To investigate the effects of RA on the activation of antioxidant enzymes, we first examined changes in the transcript abundance of the above antioxidant genes in RA- or dH_2_O-pretreated plants in the absence or presence of high-temperature stress. While exogenous RA application did not significantly change the transcript abundance of these antioxidant genes in the absence of stress, RA treatment further increased the transcript levels of APX, CAT, DHAR, and GR by 160%, 140%, 98%, and 69%, respectively (Figure 2a). Similar to the gene expression results, the high temperature-induced enzyme activities of APX, CAT, DHAR, and GR were further promoted by exogenous RA treatment in tomato plants under high-temperature conditions (Figure 2b).

In addition, nonenzymatic antioxidants such as ascorbate and glutathione acted as the heart of the redox hub to scavenge excessive ROS [21]. The redox status and content of AsA and GSH determined the capability of ROS scavenging. High-temperature stress led to a significant decline in both reduced AsA and GSH contents, but exogenous RA effectively prevented these nonenzymatic antioxidants from decreasing (Figure 2c,d). Consistently, the high temperature-induced decreases in leaf AsA to dehydroascorbate (DHA) and GSH to glutathione disulfide (GSSG) were largely prevented by RA pretreatment (Figure 2e,f). Overall, these results strongly suggested RA enhanced plant thermotolerance by activating the cellular antioxidant system.

### 2.3. Exogenous RA Treatment Promotes HSPs in Tomato Plants in Response to High-Temperature Stress

**Figure 3:**
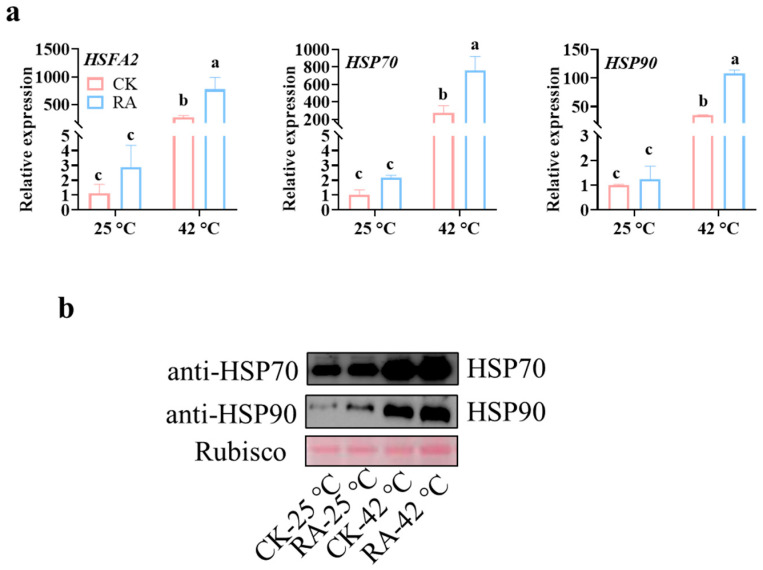
RA treatment promotes the transcription and protein abundance of tomato HSPs in response to high temperatures. (**a**) Effects of RA treatment on transcript abundance of HsfA2, HSP70, and HSP90 in tomato plants subjected to normal temperature (25 °C) or high temperature (42 °C) for 1 h. (**b**) Effects of RA treatment on the protein abundance of HSP70 and HSP90 in tomato plants with 6 h of different temperature treatments. The data presented are the mean values ± SD, *n* = 3. Statistically significant differences between treatments (*p* < 0.05, Tukey’s test) are shown by different letters.

High-temperature stress induced a variety of functional proteins denaturation and then caused irreversible damage to plants. As molecular chaperones, HSPs protected cellular proteins by preventing denatured protein aggregation and facilitating the refolding of proteins [3]. The transcript abundance of HSP genes was governed by the transactivator Hsfs by binding the heat stress elements of the HSP promoters [22]. To unravel the effects of RA on the response of Hsf and HSPs, we analyzed the transcription of some key genes to high temperature using a qRT-PCR assay. As shown in Figure 3a, the transcript abundance of the high temperature-responsive genes HsfA2, HSP70, and HSP90 were further significantly induced by exogenous RA treatment (Figure 3a). In addition, we evaluated the endogenous protein abundance of HSP70 and HSP90 using specific antibodies. Similarly, the high temperature-induced increases in HSP70 and HSP90 proteins were highly promoted by RA treatment (Figure 3b). The evidence described above indicated the activation of HSPs played a crucial role in RA-induced plant thermotolerance.

### 2.4. WRKYs Are Involved in RA-Mediated Plant Thermotolerance

**Figure 4:**
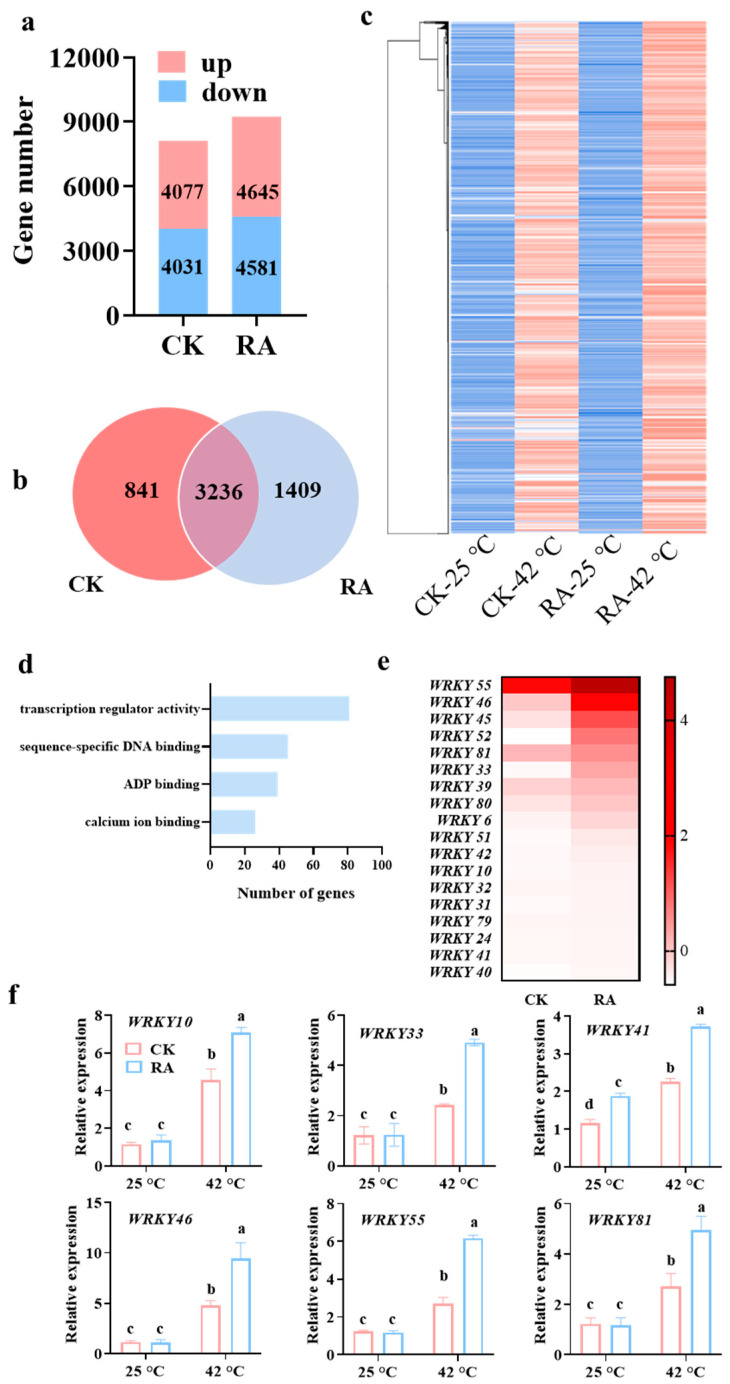
RA globally regulates high temperature-responsive gene expression in tomato plants. (**a**) Numbers of differentially high temperature-changed (fold change ≥ 2, *p* < 0.05) genes in RA-pretreated or dH_2_O-pretreated tomato plants, respectively. The color gradient scale on the right indicates the relative expression of each gene represented by each color. (**b**) Venn diagram exhibiting the numbers of high temperature-induced genes (fold change ≥ 2, *p* < 0.05) in RA-pretreated and dH_2_O-pretreated tomato plants. (**c**) Heatmap of high temperature-induced genes (fold change ≥ 2, *p* < 0.05) in RA-pretreated and dH_2_O-pretreated tomato plants. (**d**) GO analysis of RA-induced high temperature-responsive genes. (**e**) Heatmap of the induction fold of WRKY genes by high temperature in RA-pretreated and dH_2_O-pretreated tomato plants. (**f**) RT-qPCR analysis confirming transcript abundance of selected WRKY genes. The data presented in (**f**) are the mean values ± SD, *n* = 3. Statistically significant differences between treatments (*p* < 0.05, Tukey’s test) are shown by different letters.

To further investigate the global effects of RA on plant thermotolerance, we carried out an RNA-seq analysis to recognize the differential high temperature-responsive genes in response to RA or dH_2_O treatment. Of the 34,931 detected tomato transcripts, a total of 9226 (4645 up-regulated and 4581 down-regulated) and 8108 (4077 up-regulated and 4031 down-regulated) genes showed significantly differential expression (fold change ≥ 2, *p* < 0.05) within RA- and dH_2_O-treated tomato plants following high-temperature treatment, respectively (Figure 4a; Supplementary Table S1). In total, 3236 genes were found that increased in abundance in plants pretreated with either RA or dH_2_O, while other 1409 genes were only induced by high temperature in plants pretreated with RA (Figure 4b). Based on the heatmap of all high temperature-induced genes, the level of transcript abundance was globally enhanced in the RA-pretreated plants compared to dH_2_O-pretreated plants (Figure 4c). Among the high temperature-induced genes, there were an additional 3208 transcripts that showed significantly higher expression (fold change ≥ 2) in tomato plants following RA-treatment compared with that following dH_2_O-treatment under high-temperature conditions (Supplementary Table S2). Therefore, these 3208 genes were annotated as RA-induced high temperature-responsive genes. Then, the enrichment analysis of Gene Ontology (GO) categories was performed within these RA-induced high temperature-responsive genes. As shown in Figure 4d, RA-mediated transcriptional regulation was highly associated with transcription regulator activity, sequence-specific DNA binding, ADP binding, and calcium ion binding (Supplementary Table S3). Interestingly, 18 out of 81 genes (22%) from the largest cluster of transcription regulator activity were WRKY transcription factors (Figure 4e). Additionally, qRT–PCR analysis also confirmed that RA application induced a higher level of transcript abundance in *WRKY10*, *WRKY33*, *WRKY41*, *WRKY46*, *WRKY55*, and *WRKY81* in tomato plants subjected to high-temperature conditions (Figure 4f). Therefore, we implied that WRKYs played an important role in RA-mediated plant thermotolerance.

## 3. Discussion

RA is a major phenolic compound in tomatoes, of which the concentration levels ranged from 1.21 to 22.67 μg/g dry weight according to different varieties of tomatoes [16]. In some commercial cultivars of tree tomato (*Solanum betaceum* Cav.), the RA concentration reached up to 1.2 mg/g dry weight [23]. RA has a number of interesting biological activities in plant physiological processes. In our previous study, we reported that exogenous RA application could delay tomato fruit ripening by inhibiting ripening-associated ethylene production and modulating cellular redox homeostasis [24]. Treating with RA nanoparticles substantially reduced the disease severity of tomato fruits infected with phytopathogenic fungi *Alternaria alternata* and *Penicillium digitatum* during postharvest storage [25]. In addition, plant-derived RA was identified as a mimic of bacteria quorum-sensing molecule homoserine lactone, enabling strategic disruption of bacterial communication [26]. Aside from the effective function of RA on plant-microbe interaction, in the present study, we found that RA also plays a positive role in high-temperature stress tolerance. Similarly, a previous study suggested induced accumulation of phenolic compounds was associated with plant tolerance to environmental stresses [27].

Due to global warming, the threat of extremely high temperature to plant quality and yield is a world issue. When plants undergo high-temperature stress, changes in plasmalemma permeability induce osmolytes as a part of plant defense systems to effectively protect plants from stressful conditions. Osmolytes are low-molecular-weight biological compounds, including plant hormones, amino acids, sugars, methylamines, and polyphenol [28]. A series of studies showed that exogenous application of osmolytes alleviated the high temperature-induced plant wilting and enhanced the plant tolerance to high-temperature stress. Here, we reported that RA application increased tomato plant tolerance to high temperatures by protecting the plant photosystem from the damage of high-temperature stress (Figure 1a–c). Meanwhile, activation of phenolic biosynthesis and inhibition of phenolic oxidation was observed in the thermotolerance mechanism. Besides the phenolic compounds, the amino acid proline and γ-aminobutyric acid also could improve plant thermotolerance by activating the antioxidant system to alleviate high temperature-associated oxidative damage [29,30]. Exogenous trehalose effectively protected the chloroplast proteins in wheat plants to increase the photosynthetic capacity under high-temperature stress [31]. Exogenous methyl jasmonate (MeJA) strengthened the thermotolerance of perennial ryegrass (*Lolium perenne*) by modulating JA-responsive gene expression [32]. Interestingly, an appropriate concentration of MeJA efficiently induced RA accumulation via activating the gene expression associated with the RA synthetic pathway in hairy root cultures of *Prunella vulgaris* [33].

High-temperature stress is always connected with oxidative damage due to excessive ROS accumulation. Importantly, ROS presents a double-edged sword in plants against high-temperature stress. At low concentrations (30 ppm), H_2_O_2_ could enhance antioxidant enzyme activities, protect chlorophyll from degradation, and improve the boll weight and fiber quality of cotton [34]. However, high concentrations of ROS cause cellular damage. As a phenolic antioxidant, RA itself has the capability to scavenge free radicals and chelate prooxidant ions, which is mainly dependent on the number of hydroxyl groups. It is reported that the antioxidant activity of RA is stronger than tocopherol [35]. In this study, we found that RA inhibited high temperature-induced lipid peroxidation, and suppressed ROS over-accumulation (Figure 1d–f). In addition, RA also activated the cellular antioxidant system in plants. RA application further increased the transcript abundance and enzyme activity of enzymatic antioxidants, such as APX, CAT, DHAR, and GR, in response to high temperatures (Figure 2a,b). Moreover, RA protected the reduction status of nonenzymatic antioxidants from being oxidized by high-temperature stress, keeping cellular redox homeostasis during stress conditions (Figure 2c). Similarly, the majority of exogenous osmolytes application enhancing thermotolerance is highly associated with the activation of the antioxidant system. Exogenous application of brassinosteroids, 5-aminolevulinic acid, or citric acid significantly improved the activity of antioxidant enzymes and inhibited ROS production, which eventually strengthened plant thermotolerance [36,37,38].

Aside from excessive ROS scavenging, transcription regulation played a central role in the plant’s high-temperature response and induction of thermotolerance. Based on the RNA-seq data, RA-mediated thermotolerance was highly associated with transcription regulator activity, which implied the promoted effects of RA on the transcript abundance of high temperature-responsive genes of plants in response to high-temperature stress (Figure 4). As we know, when plants were subjected to high-temperature stress, a series of transcription factors would be rapidly induced. For example, HsfA2 plays a fundamental role in the Hsf-mediated high temperature-responsive transcriptional regulation network [39]. Here, we showed that exogenous RA application leads to a significant increase in *HsfA2* expression, as well as *HSP70* and *HSP90* (Figure 2a). The transcription of *HSP70* and *HSP90* was largely dependent on the activation of *HsfA2* [39]. Consistent with gene expression, the protein abundance of HSP70 and HSP90 was also promoted by RA treatment, especially in high-temperature conditions (Figure 3b). As the most abundant HSPs in the eukaryotic cell, HSP70 and HSP90 play a dual function, which not only function as molecular chaperons to inhibit protein misfolding and degradation, but also participated in signal transduction in high-temperature response [40,41]. Besides the Hsf-mediated HSP activation, we also found WRKY transcription factors were also involved in RA-mediated thermotolerance (Figure 4). An increasing number of studies indicated that the gene expression of *WRKY* transcription factors positively responded to high temperatures. Silencing high-temperature-responsive *WRKY40* in pepper plants impaired thermotolerance [42]. Moreover, WRKY40-mediated thermotolerance in pepper relied on the activation of WRKY27b via its phosphorylation by the CDPK29 protein kinase [43]. Similar to RA application, exogenous treatment of the ethylene precursor 1-aminocyclopropane-1carboxylic acid (ACC) induced gene expression of *WRKY25*, *WRKY26*, and *WRKY33*, resulting in a degree of increased thermotolerance in Arabidopsis [44]. However, not all high-temperature-induced *WRKY* genes played a positive role in thermotolerance. Silencing high-temperature-induced *WRKY27* in pepper contributed to a strengthened tolerance of high-temperature stress [45]. Therefore, the function of RA-induced high temperature-responsive WRKY genes in thermotolerance should be investigated in future work.

In summary, the data presented here reveal a novel function for RA in the thermotolerance of tomato plants. Prior to initiation of thermotolerance, exogenous RA treatment activated the antioxidant system, the protein accumulation of molecular chaperone HSPs, and the high temperature-responsive transcriptional regulation. However, the exact mechanism of how the endogenous RA metabolic pathway in tomato plants responds to high-temperature stress needs further studies.

## 4. Materials and Methods

### 4.1. Plant Material and Growth Condition

The tomato (*Solanum lycopersicum* L. cv. Condine Red) was used as test material in this study. Tomato seeds were incubated in a 50-hole plug tray covered with turf soil at 25 °C. after a 2-week germination time, individual tomato seedling was then transferred to one plastic pot filled with a sterile 7:3 (*v*/*v*) mixture of peat and vermiculite and subjected to the following controlled growth conditions: temperatures of 25 °C/21 °C (day/night), a photoperiod of 12 h/12 h (day/night), a photosynthetic photon flux density of 400 μmol m^−2^ s^−1^, and relative humidity of approximately 60%.

### 4.2. RA Pretreatment and Temperature Treatments

RA powder was purchased from Aladdin (China) and diluted by dH_2_O to a working solution of 2 mmol L^−1^. For the exogenous RA application assay, both the abaxial and the adaxial surfaces of the leaves of 5-week-old tomato plants were uniformly treated with foliar sprays of 2 mmol L^−1^ RA or dH_2_O as a control twice per d for three days before different temperature treatments.

For in planta temperature treatments, the pretreated plants were placed in growth chambers and exposed to high (42 °C) or normal temperature (25 °C). The other environmental parameters except for temperature in the growth chambers were maintained as previous growth conditions.

### 4.3. Thermotolerance and ROS Analysis

After different temperature treatments, the plant chlorophyll fluorescence was measured with a chlorophyll fluorometer (IMAG-MAXI; Heinz Walz GmbH, Effeltrich, Germany). The maximum quantum yield of PSII (Fv/Fm) was analyzed as previously described [14]. For the electrolyte leakage analysis, leaf samples were rinsed with dH_2_O three times and maintained at room temperature (25 °C) for 2 h after 10 min of vacuum infiltration. Then, the electrical conductivity (EC1) was measured. After the leaf samples were boiled at 95 °C for 20 min, the electrical conductivity (EC2) was measured when the solution cooled to room temperature [46]. The relative electrical conductivity (REC) was defined as REC (%) = EC1/EC2 × 100. The malondialdehyde (MDA) content was determined by measuring the absorbance at 532 nm by the TBA method [47].

To evaluate the accumulation of ROS, H_2_O_2,_ and O_2_^•−^ were measured using 3,3′-diaminobenzidine (DAB) and nitro blue tetrazolium (NBT) staining according to the methods of Hu [48]. For DAB staining, the fresh tomato leaves were incubated in the DAB solution (1 mg mL^−1^ of DAB in 50 mM Tris-HCl, pH 3.8) in the dark for 6 h at room temperature. For NBT staining, the fresh tomato leaves were incubated in the NBT solution (0.1 mg mL^−1^ of NBT dissolved in 25 mM HEPES, pH 7.8) for 6 h at room temperature. The stained leaves were subsequently decolorized in 95% ethanol at 95 °C for 20 mins and mounted in lactic acid/phenol/dH_2_O (1:1:1, *v*/*v*/*v*) before imaging.

### 4.4. Determination of Antioxidant Enzyme Activity and Antioxidant Contents

According to the previous methods [14], 0.3 g of plant samples was collected, added to 3 mL of PBS buffer [50 mM phosphate-buffered saline (pH 7.8), 0.2 mM EDTA, 2 mM AsA, and 2% (*w*/*v*) polyvinylpyrrolidone], ground into a homogenate in an ice bath, and then centrifuged at 12,000 rpm for 20 min at 4 °C, and the supernatant was removed. For the determination of antioxidant enzyme activity, catalase (CAT) activity was measured according to the change in absorbance value at 240 nm within 3 mins, dehydroascorbate reductase (DHAR) activity was calculated according to the decrease in absorbance at 290 nm, and the increase at 265 nm, and glutathione reductase (GR) activity was determined according to the rate of decrease at 340 nm. The GR activity and ascorbate peroxidase (APX) activity were determined by the UV absorption method.

To determine the antioxidant concentrations, 0.3 g of fresh leaf samples were ground into a powder and extracted in 2 mL of 0.2 M HCl. Afterward, the mixture was centrifuged at 12,000× *g* at 4 °C for 10 min. Then, 0.1 mL of 0.2 M phosphate-buffered saline buffer (pH 5.6) was added to 0.5 mL of the supernatant (pH 4–5), which was used to measure both the AsA and GSH through photometric assays [49].

### 4.5. Plant Total RNA Extraction and qRT–PCR Measurements

The extraction of plant total RNA was performed in accordance with the operation steps of the Plant Total RNA Extraction Kit (Aikerui Biological, Changsha, China). Then, the synthesis of cDNA was performed in accordance with the operation steps of the ReverTra Ace qRCP RT Kit (Toyobo, Osaka, Japan). qRT-PCR was carried out on a fluorescence quantitative PCR Light Cycler 480II platform (Roche). The reaction conditions were the same as those in the instructions of an AceQ qPCR SYBR Green Master Mix Fluorescent Dye Kit (Vazyme), which we used. Primer 5.0 software was used for the design of qRT–PCR primers. The sequences of the specific primers used are shown in Table 1. The internal reference gene *Actin* of tomato was selected as the internal reference index for fluorescence quantification, and the relative gene expression was calculated according to the 2^−ΔΔCT^ method.

### 4.6. Western Blot Analysis

For protein extraction, 0.3 g leaf sample was ground to powder in liquid nitrogen, and then homogenized with 2 mL extraction buffer (5 mM EDTA, 10 mM DTT, 10 mM Na_3_VO_4_, 10 mM NaF, 50 mM β-glycerophosphate, 1 mM PMSF, 100 mM HEPES, pH 7.5). After centrifugation at 13,000× *g* for 10 min, 100 μL of the supernatants were transferred to clean tubes supplemented with 20 μL 5× SDS loading buffer (5% SDS, 50% glycerol, 0.05% bromophenol blue, 225 mM Tris-HCl, pH 6.8). The extracted soluble proteins were denaturized by boiling before western blot analysis. The HSP proteins were detected by immunoblot analysis with specific antibodies against HSP70 (Agrisera, Vännäs, Swedish, AS08371) and HSP90-1 (Agrisera, Vännäs, Swedish, AS08346). The secondary antibody used subsequently was goat anti-rabbit horseradish peroxidase (HRP)-linked antibody (Cell Signaling Technology, Danvers, MA, USA, 7074).

### 4.7. RNA-Seq Analysis

Three independent repeats from RA and H_2_O pretreated five-week-old plants were performed for RNA-seq analysis. After 1-day growth under normal temperature (25 °C) or high temperature (42 °C), the leaves samples were collected. The extraction of plant total RNA was performed in accordance with the operation steps of the Plant Total RNA Extraction Kit (Aikerui Biological, Changsha, China). After the quality test, the cDNA library was constructed using Illumina’s NEBNext^®^ UltraTM RNA library ion kit, and then use the Illumina sequencing platform for transcriptome analysis by Nuohezhiyuan Technology Company (Beijing, China).

### 4.8. Statistical Analysis

The test results are the average of 3 replications. Significant difference analysis was performed by SPSS. ANOVA and Tukey’s test was used to calculate the means, and graphs were constructed by GraphPad Prism.

## 5. Conclusions

The data presented here indicate that exogenous RA application enhances thermotolerance in tomato plants via promoting an antioxidant system, high temperature-responsive transcription regulation, and HSPs accumulation. To sum up, this study not only provides an underlying mechanism of RA-mediated thermotolerance in tomato plants, but also supplies a promising strategy that can potentially protect crops from high-temperature stress in global warming future.

## Figures and Tables

**Table 1:** Gene-specific primers designed for qRT-PCR analysis.

Gene Name	Gene ID	Forward Primer, 5′-3′	Reverse Primer, 5′-3′
*Actin*	Solyc03g078400	TGTCCCTATTTACGAGGGTTATGC	CAGTTAAATCACGACCAGCAAGAT
*APX*	Solyc11g018550	CGCCATATCACACAAGAAGC	TAACTCAGAGCCACCACTGC
*GR*	Solyc09g065900	GATGATGAAATGCGAGCTGT	TTGTGTTAGGGAGACGACCA
*DHAR*	Solyc05g054760	CCCTGATGTCCTTGGAGACT	AAGAACCATTTGGGCTTGTC
*CAT*	Solyc12g094620	TGATCGCGAGAAGATACCTG	CTTCCACGTTCATGGACAAC
*HSP90*	Solyc06g036290	TGTGGGTTTCTACTCTGCGT	CTGCCCAATTGCTCTCCATC
*HSP70*	Solyc09g010630	CAAGCTGAAAGAGCTCAAGG	CTGTCCCAGCTGCATTACTT
*HsfA2*	Solyc09g082670	TCTGTTGTGACAGCAAATGG	TACTTCCTCTGCTGCTCGAT
*WRKY10*	Solyc12g096350	TGGCTGAAGACGGAGGGATA	ACGTTTGAAGCCATAGGGATCT
*WRKY33*	Solyc09g014990	CCAAACCGAGACTCGTCCAA	CGAATCCTGTGGTGCTCTGT
*WRKY41*	Solyc01g095630	ATTGGGAGCGGAGGAGTTTG	ACGATGGAGAAGACGAACCC
*WRKY46*	Solyc08g067340	GCACGCATCGATTCACACAA	CCACAACCAATCCTGTCCGA
*WRKY55*	Solyc04g072070	CCGTTGATGGTGGTGGAGAA	TCTTGGCCGGGCAATTGTAT
*WRKY81*	Solyc09g015770	GGTCAAGTCGCCGGAAGATT	AACATCGGGCGAGGTCATAC

## Data Availability

The data presented in this study are available on request from the corresponding author.

## Supplementary Materials

The following supporting information can be downloaded at: https://www.mdpi.com/article/10.3390/plants11091172/s1, Table S1: High temperature-responsive genes in tomato; Table S2: The RA-induced higher temperature-responsive genes in tomato plants; Table S3: List genes of different GO categories analyzing with RA-induced high temperature-responsive genes.
